# Large-scale exome datasets reveal a new class of adaptor-related protein complex 2 sigma subunit (AP2σ) mutations, located at the interface with the AP2 alpha subunit, that impair calcium-sensing receptor signalling

**DOI:** 10.1093/hmg/ddy010

**Published:** 2018-01-09

**Authors:** Caroline M Gorvin, Raghu Metpally, Victoria J Stokes, Fadil M Hannan, Sarath B Krishnamurthy, John D Overton, Jeffrey G Reid, Gerda E Breitwieser, Rajesh V Thakker

**Affiliations:** 1 Academic Endocrine Unit, Radcliffe Department of Medicine, Oxford Centre for Diabetes, Endocrinology and Metabolism (OCDEM), University of Oxford, Oxford OX3 7LJ, UK; 2 Geisinger Clinic, Weis Center for Research, Danville, PA 17822, USA; 3 Department of Musculoskeletal Biology, Institute of Ageing and Chronic Disease, University of Liverpool, L7 8TX UK; 4 Regeneron Genetics Center, Tarrytown, NY 10591, USA

## Abstract

Mutations of the sigma subunit of the heterotetrameric adaptor-related protein complex 2 (AP2σ) impair signalling of the calcium-sensing receptor (CaSR), and cause familial hypocalciuric hypercalcaemia type 3 (FHH3). To date, FHH3-associated AP2σ mutations have only been identified at one residue, Arg15. We hypothesized that additional rare AP2σ variants may also be associated with altered CaSR function and hypercalcaemia, and sought for these by analysing >111 995 exomes (>60 706 from ExAc and dbSNP, and 51 289 from the Geisinger Health System-Regeneron DiscovEHR dataset, which also contains clinical data). This identified 11 individuals to have 9 non-synonymous AP2σ variants (Arg3His, Arg15His (x3), Ala44Thr, Phe52Tyr, Arg61His, Thr112Met, Met117Ile, Glu122Gly and Glu142Lys) with 3 of the 4 individuals who had Arg15His and Met117Ile AP2σ variants having mild hypercalcaemia, thereby indicating a prevalence of FHH3-associated AP2σ mutations of ∼7.8 per 100 000 individuals. Structural modelling of the novel eight AP2σ variants (Arg3His, Ala44Thr, Phe52Tyr, Arg61His, Thr112Met, Met117Ile, Glu122Gly and Glu142Lys) predicted that the Arg3His, Thr112Met, Glu122Gly and Glu142Lys AP2σ variants would disrupt polar contacts within the AP2σ subunit or affect the interface between the AP2σ and AP2α subunits. Functional analyses of all eight AP2σ variants in CaSR-expressing cells demonstrated that the Thr112Met, Met117Ile and Glu142Lys variants, located in the AP2σ α4-α5 helical region that forms an interface with AP2α, impaired CaSR-mediated intracellular calcium (${\text{Ca}}_{i}^{2\text{+}}$) signalling, consistent with a loss of function, and this was rectified by treatment with the CaSR positive allosteric modulator cinacalcet. Thus, our studies demonstrate another potential class of FHH3-causing AP2σ mutations located at the AP2σ-AP2α interface.

## Introduction

Familial hypocalciuric hypercalcaemia (FHH) is an autosomal dominant condition characterized by lifelong mild-to-moderate elevations of serum calcium concentrations in association with normal or mildly raised serum parathyroid hormone (PTH) concentrations and low urinary calcium excretion (1,2). FHH is genetically heterogeneous and at present comprises three reported subtypes (FHH1–3). FHH1 (OMIM #145980) is due to heterozygous loss-of-function mutations affecting the G-protein-coupled calcium-sensing receptor (CaSR), encoded by the *CASR* gene, and FHH2 (OMIM #145981) is due to heterozygous loss-of-function mutations of the G-protein alpha-11 subunit (Gα_11_), encoded by the *GNA11* gene (3,4). The CaSR and Gα_11_ play a critical role in systemic calcium homeostasis by detecting alterations in extracellular calcium (${\text{Ca}}_{\;e}^{2\text{+}}$) concentrations and initiating multiple intracellular signalling cascades that include phospholipase-C-mediated accumulation of inositol 1,4,5-trisphosphate (IP_3_), and increases in intracellular calcium (${\text{Ca}}_{\;i}^{2\text{+}}$) concentrations (5,6), which in turn leads to decreases in PTH secretion and increases in urinary calcium excretion (7). 

**Figure 1.**
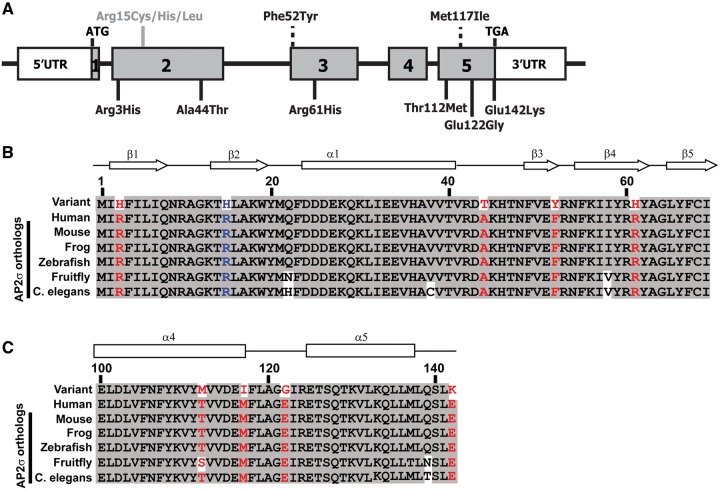
Schematic representation of *AP2S1* gene showing locations of the identified variants. (**A**) Representation of the genomic organization of the human *AP2S1* gene showing the location of the identified variants. The *AP2S1* gene consists of 5 exons (shaded) with the start (ATG) and stop (TGA) codons located in exons 1 and 5, respectively. Untranslated regions are represented by open boxes. The Arg (R)15 residue (indicated in grey with a solid line), at which the previously reported FHH3-associated mutations of Cys (C), His (H) and Leu (L) have been identified (18), is located in exon 2. Two novel AP2σ variants, Phe52Tyr (F52Y) and Met117Ile (M117I), identified in the DiscovEHR cohort are located within exons 3 and 5, respectively (indicated by a broken line above the exons). The six AP2σ variants [Arg3His (A3H), Ala44Thr (A44T), Arg61His (R61H), Thr112Met (T112M), Glu122Gly (E122G) and Glu142Lys (E142K)] identified in the ExAc and dbSNP databases are located in exons 2, 3 and 5, and are shown (by solid lines) below the exons. (**B–C**) Multiple protein sequence alignment of residues comprising (B) the β1**–**β5 strands and the α1 helix (residues 1**–**69) and (C) the α4 and α5 helices (residues 107**–**142) of AP2σ-subunit orthologs encoded by *AP2S1* exons 2, 3 and 5. Conserved residues are shaded grey. The WT and variant residues are shown in red. The FHH3-associated R15H mutation is shown in blue. The R3, A44, F52, R61, T112, M117, E122 and E142 residues are evolutionarily conserved, thereby indicating that they likely have important structure**–**function roles in AP2σ.

Familial hypocalciuric hypercalcaemia type 3 (FHH3) (OMIM #600740) represents a clinically more severe form of FHH, which may be associated with symptomatic hypercalcaemia, osteoporosis, osteomalacia and cognitive dysfunction (8,9). FHH3 is caused by mutations of the adaptor-related protein complex 2 (AP2) sigma subunit (AP2σ), encoded by the *AP2S1* gene which consists of five exons (Fig. 1). AP2, which is a ubiquitously expressed heterotetrameric protein comprising α, β, µ and σ subunits (10) (Supplementary Material, Fig. S1), plays a fundamental role in the clathrin-mediated endocytosis of G-protein-coupled receptors (GPCRs) such as the CaSR. The AP2 complex operates as two heterodimers, one comprised of the AP2α and AP2σ subunits, and the other the AP2β and AP2µ subunits (11–13) (Supplementary Material, Fig. S1). In the inactive state, the AP2α and AP2β subunits form a diamond-shaped outer complex, and the AP2µ and AP2σ subunits, are buried within the core of the AP2 complex (10–12,14–16). Upon activation, AP2α and AP2β bend away from each other, with the AP2σ subunit accompanying AP2α and the AP2µ subunit being displaced toward the plasma membrane (11,12,14). In this open conformation, the AP2µ and AP2σ subunits bind to endocytic motifs on transmembrane cargo proteins, and thereby facilitate the association of clathrin with the AP2 complex (12,14,17).

**Figure 2.**
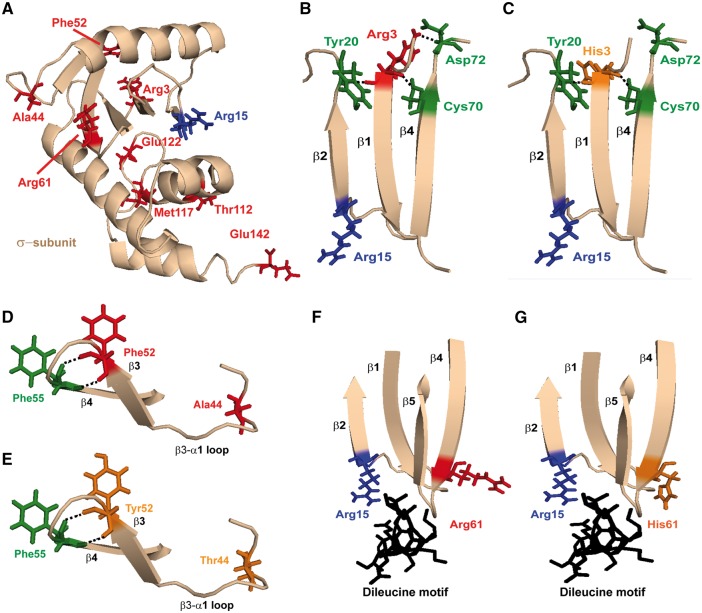
Structural characterization of the AP2σ variants encoded by *AP2S1* exons 2 and 3. (**A**) Close-up view of the AP2 σ-subunit with the residues having variants within the ExAc (Arg3His, Arg61His, Thr112Met, Glu122Gly and Glu142Lys), dbSNP (Ala44Thr) and DiscovEHR (Phe52Tyr and Met117Ile) cohorts shown in red. The FHH3 mutations affect the Arg15 residue (shown in blue). (**B**) The AP2σ Arg3 residue is located within the β1 strand that lies adjacent to the β2 strand in which the FHH3-associated Arg15 residue is located. Arg3 forms polar contacts with residues Tyr20, Cys70, Asp72 on the adjacent β2-α1 loop, β4-strand and β4-β5 loop, respectively. (**C**) Mutation of the Arg3 residue to His3 leads to loss of the polar contact with Asp72, which may disrupt the stability of the σ-subunit. (**D**) The Ala44 residue is located in the α1-β3 loop, and the Phe52 residue is located distal to this, in the β3 strand. Ala44 is not predicted to form polar contacts with any neighbouring residues, while Phe52 is predicted to form polar contacts with the adjacent Phe55 located in the β4 strand. (**E**) Mutation of the Ala44 and Phe52 residues to Thr44 and Tyr52, respectively, are not predicted to disrupt or form new contacts with other AP2σ residues. (**F**) The Arg61 residue is located in the β4 strand that lies within a cluster of β-strands that converge close to the Arg15 AP2σ residue and the dileucine motif binding site (black) of cargo proteins, such as the CaSR and other GPCRs. (**G**) Mutation of Arg61 to His61 may affect binding to the cargo protein dileucine recognition motif.

All three FHH3-associated mutations reported to date involve the AP2σ Arg15 residue (8,18–22), which is located within the β2-strand (Fig. 2), and each of the three different missense mutations (Arg15Cys, Arg15His or Arg15Leu) (Fig. 1), are postulated to disrupt polar contacts between the AP2σ Arg15 residue and the dileucine motif within the intracellular domain of the CaSR, which likely targets it for endocytosis (8,10,18). Indeed, these three FHH3-associated AP2σ Arg15 mutations have been shown to alter CaSR cell-surface expression and to have a dominant-negative effect on CaSR-mediated signalling (8,18). In contrast, other potential mutations of the AP2σ Arg15 residue (Arg15Gly, Arg15Pro or Arg15Ser) have not been observed in humans, and *in vitro* studies have shown these mutations to impair cell growth (8). These findings indicate that potential mutations affecting the *AP2S1* gene, which is highly conserved in zebrafish, fruitfly and yeast homolog proteins with >99%, >96% and >95% amino acid identity, respectively, may not be commonly observed as they affect cellular viability (13). Moreover, the large exome and genome datasets contain *AP2S1* variants at very low frequencies. For example, examination of the >13 000 alleles from the exome variant server did not reveal the presence of any AP2σ variants, whilst the 1000Genomes and Exome Aggregation Consortium (ExAc) databases (23,24) contained only six coding variants, and the dbSNP database had only one AP2σ missense variant. The pathophysiological significance of these rare coding AP2σ variants is unknown, especially as these large sequencing projects do not contain phenotype information on individuals. Thus, these rare coding AP2σ variants may be benign polymorphisms, and we have previously shown that some AP2σ variants do not alter CaSR signalling or result in an abnormal phenotype. For example, our *in vitro* examination of *N*-ethyl-*N-*nitrosourea (ENU) induced AP2σ variants in mice demonstrated that two missense AP2σ variants, Tyr20Asn and Ile123Asn, had no effect on CaSR signalling, and that mice heterozygous for a donor splice-site variant, which results in an in-frame deletion of 17 amino acids, had normal serum and urinary calcium, despite a >50% reduction in AP2σ protein expression (25). This emphasises the need for *in vitro* and *in vivo* functional assessments of AP2σ variants, in determining their potential role in the pathophysiology of calcium homeostasis.

The DiscovEHR exome sequencing dataset, which has arisen from a collaboration between the Regeneron Genetics Center and Geisinger Health System (26), offers a new opportunity for studying the role of rare coding variants in human pathophysiology, as the dataset contains matched genotype and phenotype information from 51 289 individuals (26). We therefore investigated the DiscovEHR dataset, as well as the ExAc and dbSNP datasets, to identify rare coding AP2σ variants, and characterized their functional and clinical consequences. These studies demonstrated that AP2σ variants located at the interface between the AP2σ and AP2α subunits were associated with impaired CaSR signalling and hypercalcaemia.

## Results

### Identification of two AP2σ variants associated with hypercalcaemia in the DiscovEHR exome sequencing dataset

An analysis of the DiscovEHR exome sequencing dataset, which at the time of investigation contained the exomes from 51 289 adult patients (26), revealed five females to have heterozygous coding *AP2S1* variants (Supplementary Material, Table S1, Fig. S1). Three of these patients harboured the reported FHH3-causing Arg15His mutation located in exon 2 of *AP2S1* (Fig. 1) (18), and two of these three patients were found to have mild hypercalcaemia (Table 1). The other two patients had novel variants which comprised: a heterozygous G-to-A transition at nucleotide c.155, located in exon 3, leading to substitution of the wild-type (WT) phenylalanine (Phe) with the mutant tyrosine (Tyr) at residue 52 of the AP2σ protein; and a heterozygous G-to-T transversion at nucleotide c.350, located in exon 5, resulting in a missense substitution of the WT methionine (Met) to a mutant isoleucine (Ile) at residue 117 of the AP2σ protein (Fig. 1, Supplementary Material, Table S1). The novel Phe52Tyr and Met117Ile variants, which were observed only once in the DiscovEHR dataset and were absent in the ExAc dataset, affected evolutionarily conserved AP2σ residues (Fig. 1), and assessments using the SIFT and Polyphen-2 prediction software (27,28) revealed the following. SIFT predicted that both of these variants would be disease-causing or damaging, whilst Polyphen-2 predicted that Phe52Tyr would be tolerated, but that the Met117Ile was likely to be damaging (Supplementary Material, Table S1). The Polyphen-2 predictions were found to be in agreement with the results of the serum calcium concentrations obtained from the Geisinger Health System electronic health records (Table 1), which revealed that the patient with the Phe52Tyr variant was normocalcaemic, but that the patient harbouring the Met117Ile AP2σ variant had mild hypercalcaemia (Table 1). Thus, these studies reveal that 4 out of 51 289 individuals in the DiscovEHR cohort harboured AP2σ variants that were associated with hypercalcaemia and/or FHH3, thereby indicating an overall prevalence of ∼7.8 per 100 000 for disease-causing AP2σ variants in this cohort.

**Table 1.** Genotypes and phenotypes of five patients with *AP2S1* variants from the DiscovEHR cohort

Nucleotide change	Predicted change	Sex^a^	Age (years)	Total serum calcium adjusted for albumin (mg/dL)^b^^,c^	Co-existing clinical conditions
c.44G>A	Arg15His	F	72	10.06	Polycystic kidney disease, hypothyroidism, hypertension, diabetes mellitus type 2, vitamin D deficiency, osteoporosis
c.44G>A	Arg15His	F	68	10.08	Multiple sclerosis, vitamin D deficiency, osteoporosis, obesity
c.44G>A	Arg15His	F	73	9.80	Pituitary tumour, asthma
c.155G>A	Phe52Tyr	F	53	9.56	Obesity, vitamin D deficiency, hypothyroidism
c.350G>T	Met117Ile	F	34	10.10^d^	—^e^

a F, female.

b Serum calcium normal range = 8.30–10.00 mg/dL, which is defined as the mean ± 2 standard deviations (SD). Hypercalcaemia is defined as a serum calcium concentration greater than 2 SDs above the normal mean, and hypercalcaemia is generally considered to be mild, moderate or severe for serum calcium concentrations that are >10.00 mg/dL but ≤12.00 mg/dL, between 12.01 and 14.00 mg/dL, and ≥14.01 mg/dL, respectively (40). The serum calcium concentrations are adjusted or ‘corrected’ to a reference (usually the mean for the normal population) albumin concentration, because ∼50% of total serum calcium is bound to albumin, and thus variations in serum albumin concentration can affect total serum calcium concentrations (40). The formula used to derive the total serum calcium concentration adjusted for albumin is: adjusted total serum calcium concentration = measured total serum calcium + [0.8 × (4.0 – measured total serum albumin concentrations)] (40).

c The initial serum calcium value obtained from ambulatory patients in the outpatient department is shown.

d Serum albumin is not available for this patient, and the uncorrected total serum calcium concentration is shown.

e —, clinical details not available.

### Identification of six non-synonymous AP2σ variants within the ExAc and dbSNP databases

The identification of a non-Arg15 AP2σ variant that was associated with a mild elevation of serum calcium concentration (Fig. 1 and Table 1) suggested that additional rare AP2σ variants that may disrupt calcium homeostasis may also be present in exome sequence databases. We therefore searched for *AP2S1* variants in >60 706 unrelated individuals in the ExAc (24), 1000Genomes (23) and dbSNP datasets (Supplementary Material, Tables S2 and S3). This identified 27 AP2σ variants, which comprised 6 non-synonymous germline variants and 21 synonymous variants (Fig. 1, Supplementary Material, Tables S2 and S3). Variants involving the AP2σ Arg15 residue were not identified and there were also no nonsense mutations of AP2σ. This number of non-synonymous AP2σ variants was significantly lower than the expected numbers of missense (*n* = 63.7) and nonsense (*n* = 7.8) variants for a protein of 142 amino acids, estimated using the ExAc database (24) (observed AP2σ variants vs. expected gene variants: missense = 0.009% vs. 0.1%; and nonsense = 0% vs. 0.01%, χ^2^ = *P* < 0.0001). Two of the non-synonymous AP2σ variants were found in exon 2, and consisted of Arg3His (G > A at c.8) and Ala44Thr (C > T at c.130), one non-synonymous AP2σ variant Arg61His (C > T at c.182) was found in exon 3, and the remaining three non-synonymous variants were identified in exon 5, and consisted of Thr112Met (C > T at c.335), Glu122Gly (A > G at c.365) and Glu142Lys (G > A at c.424). All of these six non-synonymous variants, which were observed only once in the ExAc or dbSNP datasets and were not present in the DiscovEHR dataset, affected evolutionarily conserved residues (Fig. 1B and C), thereby, indicating they may represent pathogenic mutations rather than benign polymorphisms.

### Structural characterization of eight novel AP2σ variants

**Figure 3.**
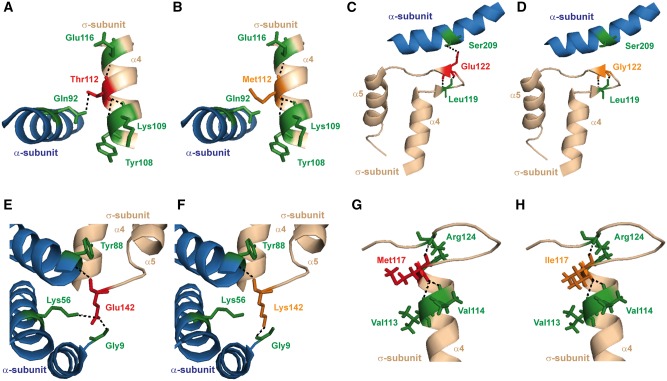
Structural characterization of the AP2σ variants within the AP2σ α4-α5 helices encoded by *AP2S1* exon 5. (**A**) Structural model of the α4-helix of the AP2σ subunit (shown in light brown) with the adjacent α5-helix of the AP2α subunit (shown in blue). The Thr112 residue forms a polar contact with the Tyr108, Lys109 and Glu116 residues on the α4-helix of AP2σ, and the Gln92 residue on the α5-helix of the AP2α subunit. (**B**) Mutation of the Thr112 residue to Met112 leads to loss of the polar contact with Gln92, and therefore may impair AP2σ**–**AP2α subunit interactions. (**C**) Structural model of the α4**–**α5 helices of the AP2σ subunit (shown in light brown) with the adjacent α12-helix of the AP2α subunit (shown in blue). The Glu122 residue is located within the α4**–**α5 loop, and forms polar contacts with Leu119 of the AP2σ subunit, and Ser209 in the α12-helix of the AP2α subunit. (**D**) Mutation of residue Glu122 to Gly122 results in loss of the contact with Ser209, and thus the Gly122 variant may impair AP2σ**–**AP2α subunit interactions. (**E**) Structural model of the α4 and α5 helices of AP2σ (shown in light brown) and the AP2α subunit (shown in blue). The Glu142 residue is located at the end of the α4-helix of the AP2σ subunit and forms polar contacts with Gly9, Lys56 and Tyr88 of the AP2α subunit. (**F**) Mutation of the Glu142 residue to Lys142 disrupts the polar contact with Lys56, and thus the Lys142 variant may impair AP2σ**–**AP2α subunit interactions. (**G**) Structural model of the α4 helix of AP2σ (shown in light brown) with the Met117 residue indicated in red. The Met117 residue forms polar contacts with Val113, Val114 and Arg124. (**H**) Mutation of the Met117 residue to Ile117 is not predicted to alter residue hydrophobicity and disrupt these polar contacts.

The predicted effects of the eight novel non-synonymous AP2σ variants, which comprised two from the DiscovEHR dataset (Phe52Tyr and Met117Ile), five from the ExAc dataset (Arg3His, Arg61His, Thr112Met, Glu122Gly and Glu142Lys) and one from dbSNP (Ala44Thr), on the structure of the AP2σ protein and their interactions with other subunits within the AP2 complex were characterized (Fig. 2). The eight AP2σ variants were found not to alter the secondary structure of the AP2σ α-helical or β-strand structures. Three-dimensional modelling of the AP2σ variants was undertaken using the reported crystal structure of the AP2 heterotetramer (12). The AP2σ subunit is comprised of five α-helices and a cluster of five β-strands (12), and the analysis of three-dimensional modelling, revealed that four of the variants were situated in the β-strand cluster, which is involved in the binding of the AP2σ subunit to the cargo protein dileucine motif (10) (Fig. 2), and the other four variants were located in the α4 and α5 helices, which lie close to the AP2α subunit, that forms a heterodimer with AP2σ (11,12) (Fig. 3 and Supplementary Material, Fig. S1). Further analysis of the four variants (Arg3His, Ala44Thr, Phe52Tyr and Arg61His) within the β-strand cluster, revealed that the Arg3His variant, which is located distal to Arg15 in the β1-strand (Fig. 2B), would likely disrupt a polar contact between the WT Arg3 residue and the Asp72 residue on the β5-α2 loop (Fig. 2C), and this would potentially impair stability of the AP2σ subunit. However, the Ala44Thr and Phe52Tyr variants, which are located in the α1-β3 loop and the β3-strand of the AP2σ subunit, respectively, were not predicted to disrupt intra- or inter-subunit interactions (Fig. 2D and E), but the Arg61His variant, which is located in the β4-strand and close to the site of the dileucine binding motif of membrane cargo proteins, was predicted to potentially disrupt the binding of AP2σ to membrane cargo proteins (Fig. 2F and G).

**Figure 4.**
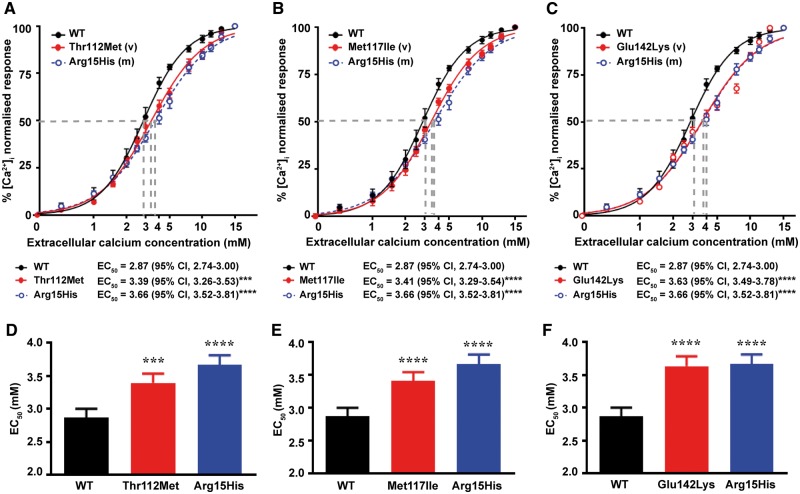
Intracellular calcium responses of cells expressing the AP2σ variants (Thr112Met, Met117Ile and Glu142Lys) encoded by *AP2S1* exon 5 and located in the AP2σ α4-α5 helices. ${\text{Ca}}_{\;i}^{2\text{+}}$ responses, measured by flow cytometry, to changes in [Ca^2+^]_e_ of HEK-CaSR cells transfected with wild-type (WT), or (**A**) Thr112Met, (**B**) Met117Ile or (**C**) Glu142Lys AP2σ variants (v), or the FHH3-associated Arg15His mutant (m) constructs. The ${\text{Ca}}_{\;i}^{2\text{+}}$ responses to changes in [Ca^2+^]_e_ are expressed as a percentage of the maximum normalized responses and shown as the mean±SEM of 4**–**8 independent transfections. The Thr112Met, Met117Ile and Glu142Lys AP2σ variants led to a rightward shift in the concentration-response curve (red line), compared to WT. Variant responses were similar to the Arg15His loss-of-function mutant (blue line). (**D–F**) Histograms showing the mean half-maximal concentration (EC_50_) with 95% confidence intervals (CI) and *P*-values of cells expressing WT (black bar), (**D**) Thr112Met, (**E**) Met117Ile or (**F**) Glu142Lys (red bars) and Arg15His (blue bar) AP2σ proteins. Statistical analysis was performed using the *F-*test. ****P < 0.0001, ***P < 0.001, compared to WT.

Similar analysis of the four variants (Thr112Met, Met117Ile, Glu122Gly and Glu142Lys) located in the α4-α5 region at the C-terminus of the AP2σ protein (Fig. 2), revealed that the Thr112Met, Glu122Gly and Glu142Lys variants, would likely disrupt AP2σ-AP2α inter-subunit interactions, and thereby impair the structural integrity of this heterodimer and/or activation of the AP2 complex (Fig. 3A–F). However, the side chain of Met117 and the variant Ile117 face away from the AP2σ–AP2α interface, and thus the Met117Ile variant was not predicted to alter interactions with the AP2α subunit, or to disrupt contacts within the AP2σ α4-helix (Fig. 3G and H). The consequences of all these predicted structural alterations resulting from the AP2σ variants, on CaSR-mediated signalling were further assessed (Fig. 4 and Supplementary Material, Figs S2 and S3).

### Effects of the eight novel AP2σ variants on CaSR-mediated signalling and treatment with cinacalcet

To determine the effects of the AP2σ variants on CaSR-mediated signalling, HEK293 cells stably expressing the CaSR (HEK-CaSR) were transiently transfected with pBI-CMV4-*AP2S1* constructs expressing either the WT or mutant AP2σ proteins, as described (8). This bidirectional pBI-CMV4 vector allowed for co-expression of AP2σ and red fluorescent protein (RFP) at equivalent levels, as reported previously (8). The expression of AP2σ and RFP was confirmed by fluorescence microscopy and/or western blot analyses (Supplementary Material, Fig. S2A and B). The expression of AP2σ was shown to be similar in cells transiently transfected with WT or variant proteins and higher than the endogenous expression of AP2σ (Supplementary Material, Fig. S2B). The ${\text{Ca}}_{\;e}^{2\text{+}}$-induced ${\text{Ca}}_{\;i}^{2\text{+}}$ responses of HEK-CaSR cells transiently expressing the AP2σ variants was assessed using a flow cytometry-based assay, as described (18). The reported FHH3-causing Arg15His AP2σ variant (18) was used as a loss-of-function control in the flow cytometry assays. The ${\text{Ca}}_{\;i}^{2\text{+}}$ responses in WT and variant AP2σ-expressing cells increased in a dose-dependent manner following exposure to increasing concentrations of ${\text{Ca}}_{\;e}^{2\text{+}}$. However, responses in cells expressing the Thr112Met, Met117Ile or Glu142Lys AP2σ variants, which are all located in the α4-α5 helical region of the AP2σ subunit (Fig. 3), were significantly reduced when compared to WT expressing cells (Fig. 4A–C), consistent with these AP2σ variants leading to loss of CaSR function (18). Thus, the Thr112Met, Met117Ile and Glu142Lys variants and Arg15His mutant expressing cells showed a rightward shift in the concentration–response curves (Fig. 4A–C), with significantly increased half-maximal (EC_50_) values (*n* = 4–8) of 3.39 mm [95% confidence interval (CI) 3.26–3.53 mm] for Thr112Met expressing cells (*P* < 0.001), 3.41 mm (95% CI 3.29–3.54 mm) for Met117Ile expressing cells (*P* < 0.001), 3.63 mm (95% CI 3.49–3.78 mm) for Glu142Lys expressing cells (*P* < 0.0001) and 3.66 mm (95% CI 3.52–3.81 mm) for Arg15His expressing cells (*P* < 0.0001), compared to 2.87 mm (95% CI 2.74–3.00 mm) for WT expressing cells (Fig. 4D–F), In contrast, cells expressing the other five AP2σ variants (Arg3His, Ala44Thr, Phe52Tyr, Arg61His and Glu122Gly) had ${\text{Ca}}_{\;i}^{2\text{+}}$ responses and EC_50_ values that were not significantly different to the WT expressing cells, indicating that they are likely to be benign polymorphisms and not mutations (Supplementary Material, Fig. S3).

**Figure 5.**
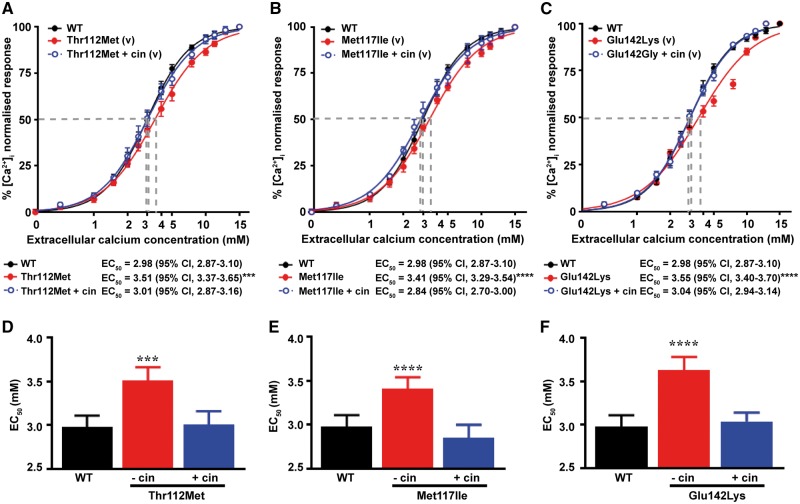
Effect of cinacalcet on intracellular calcium responses of cells expressing the AP2σ variants Thr112Met, Met117Ile and Glu142Lys. ${\text{Ca}}_{\;i}^{2\text{+}}$ responses, measured by flow cytometry, to changes in [Ca^2+^]_e_ of HEK-CaSR cells transfected with wild-type (WT) or (**A**) Thr112Met, (**B**) Met117Ile or (**C**) Glu122Gly variants (v) AP2σ proteins. The ${\text{Ca}}_{\;i}^{2\text{+}}$ responses to changes in [Ca^2+^]_e_ are expressed as a percentage of the maximum normalized responses and shown as the mean±SEM of 4**–**8 independent transfections. The Thr112Met, Met117Ile and Glu142Gly AP2σ variants led to a rightward shift in the concentration**–**response curve (red line), compared to WT, and the addition of 10 nm cinacalcet (cin) rectified these rightward shifts of the AP2σ variants (blue line). (**D–F**) Histograms showing the mean half-maximal concentration (EC_50_) with 95% confidence intervals (CI) and *P*-values of cells expressing WT (black bar), (**D**) Thr112Met, (**E**) Met117Ile and (**F**) Glu142Gly variants treated with vehicle (red bars), or treated with 10 nm cinacalcet (blue bars) AP2σ proteins. Statistical analysis was performed using the *F-*test. ****P < 0.0001, ***P < 0.001 compared to WT.

We have previously demonstrated that the elevated EC_50_ values for CaSR-mediated ${\text{Ca}}_{\;i}^{2\text{+}}$ release in cells expressing the FHH3-causing AP2σ Arg15 mutations can be rectified by treatment with cinacalcet, which is a CaSR positive allosteric modulator (29). To investigate whether the loss of function observed in HEK-CaSR cells expressing the AP2σ Thr112Met, Met117Ile or Glu142Lys mutations may also be corrected by allosteric modulation, we tested responses in the presence of 10 nM cinacalcet, a dose which normalizes altered signalling responses of the FHH3-causing Arg15 AP2σ mutations (29). Treatment with 10 nm cinacalcet, led to a leftward shift of the abnormal dose–response curves for all three variants (Fig. 5A–C), such that the EC_50_ values of AP2σ variant expressing cells were decreased and similar to values of WT AP2σ expressing cells [EC_50_ values without cinacalcet (*n* = 4–8) were 3.51 mm (95% CI 3.37–3.65 mm) for Thr112Met expressing cells (*P* < 0.001), 3.41 mm (95% CI 3.29–3.54 mm) for Met117Ile expressing cells (*P* < 0.0001) and 3.55 mm (95% CI 3.40–3.70 mm) for Glu142Lys expressing cells (*P* < 0.0001); with cinacalcet treatment, EC_50_ values (*n* = 4–8) were 3.01 mm (95% CI 2.87–3.16 mm) for Thr112Met expressing cells [*P* = not significant (ns)], 2.84 mm (95% CI 2.70–3.00 mm) for Met117Ile expressing cells (*P* = ns), and 3.04 mm (95% CI 2.94–3.14 mm) for Glu142Lys expressing cells (*P* = ns), compared to 2.98 mm [(95% CI 2.87–3.10 mm) for WT expressing cells] (Fig. 5D–F). Thus, cinacalcet is able to correct the loss of function associated with the AP2σ α4-α5 helix variants (Thr112Met, Met117Ile and Glu142Lys).

## Discussion

Our studies, which have analysed exome datasets from ∼112 000 individuals for AP2σ variants that may cause FHH3 and abnormalities of CaSR-mediated signalling, provide several new insights about: the prevalence of FHH3-associated AP2σ mutations, the structural-functional consequences of these mutations; and the importance of the AP2σ α4 and α5 helices in mediating activation of the AP2 complex that has a critical role in clathrin-mediated endocytosis of cell-surface proteins such as GPCRs. Thus, our analysis of the DiscovEHR cohort, of adult patients from a stable regional population in Pennsylvania (30), has provided the first prevalence estimate for FHH3, and found this to be ∼7.8 cases per 100 000, which is similar to the estimated prevalence of 1–9 cases per 100 000 for FHH1 (31), and in keeping with FHH being defined as a rare disease (32). Three out of the five DiscovEHR patients with rare AP2σ variants, had the Arg15His AP2σ mutation, which has been reported to cause a milder form of hypercalcaemia than the FHH3-causing Arg15Cys and Arg15Leu AP2σ mutations (8). In keeping with this, the Arg15His AP2σ mutation in the DiscovEHR cohort was associated with serum calcium concentrations that were mildly elevated, in two patients, or at the upper limit of normal in one patient (Table 1). The two patients with the Arg15His mutation, who were hypercalcaemic, also had vitamin D deficiency and/or chronic kidney disease, and this may have influenced their mild hypercalcaemia (Table 1). In addition, our results reveal that some individuals who are heterozygous for the AP2σ Arg15His may be normocalcaemic and this is similar to the reports that heterozygous loss-of-function CaSR mutations, which are associated with hypercalcaemia in the majority of patients may also rarely be associated with normocalcaemia in some individuals (33,34). Moreover, our results are the first to report that an AP2σ mutation (Met117Ile) that does not involve the Arg15 residue can impair CaSR-mediated signalling (Fig. 4), and may be associated with hypercalcaemia (Table 1), consistent with FHH3. Thus, our findings indicate that non-Arg15 AP2σ mutations may cause FHH3, and that the current practice of only searching for mutations of the AP2σ Arg15 residues in exon 2 will need to be altered to include DNA sequence analysis of the other *AP2S1* exons.

Indeed, our demonstration that mutations (e.g. Thr112Met, Met117Ile and Glu142Lys) located in the AP2σ α4-α5 helices impair the intracellular calcium signalling responses of CaSR-expressing cells (Fig. 4, Supplementary Material, Table S4) indicate the importance of this region for CaSR signalling and/or clathrin-mediated endocytosis. Our structural studies predicted that variations in the AP2σ α4-α5 helices would disrupt contacts with the AP2α subunit, and thus are likely to impair AP2 complex formation and/or the conformational changes necessary for AP2 complex activation (Fig. 3, Supplementary Material, Table S4). Indeed, in previous studies in which AP2α was deleted in *C. elegans*, the AP2σ homolog was shown to be unstable, whilst the contacts between AP2β and AP2µ (which represents the other heterodimer in the AP2 structure) were unaffected (13). Thus, these previous studies and our investigation of AP2σ α4-α5 variants suggest that mutations located at the AP2σ–AP2α interface are likely to impair heterodimer function. These studies also indicate that AP2σ mutations could be divided into two types: those impairing cargo protein recognition, as is the case for Arg15 mutations; and those impairing AP2 complex activation, as is the case for AP2σ α4-α5 mutants. In addition, our *in vitro* investigations demonstrated that the CaSR-positive allosteric modulator, cinacalcet, can normalize the impaired signalling responses associated with mutations (Thr112Met, Met117Ile and Glu142Lys) of the AP2σ α4-α5 helices, and this is similar to our previous report that cinacalcet can rectify the *in vitro* and *in vivo* abnormalities of CaSR-mediated signalling associated with AP2σ Arg15 mutants (29). Thus, cinacalcet may have therapeutic potential for patients who have such AP2σ mutations located in the α4-α5 helices, together with symptomatic hypercalcaemia or additional clinical phenotypes such as cognitive dysfunction that have been observed in patients with FHH3-causing Arg15 AP2σ mutations (8,29).

Our findings also highlight the importance of functional characterization of variants identified in large-scale sequencing databases (e.g. ExAc), to assess whether these may represent pathogenic mutations. Thus, population cohorts that contain sequencing data on patients with a range of disorders [including diabetes mellitus type 2, heart disease and inflammatory bowel disease (24)], should be used with caution as representative of the ‘normal’ population. Furthermore, in the absence of clinical data, from the ExAc and dbSNP databases, it is difficult to determine whether the carriers within the cohort are unaffected by the disorder under investigation. However, the DiscovEHR cohort, which has available both genetic and clinical data (26), allows assessment of the pathogenic effect of genetic variants. In addition, our studies illustrate the reliability and difficulties associated with the pathogenicity prediction and three-dimensional modelling programs (Supplementary Material, Table S4). Thus, the pathogenicity and three-dimensional modelling programs predicted that the AP2σ variants Thr112Met and Glu142Lys would likely be deleterious (Fig. 3 and Supplementary Material, Table S2) and that Ala44Thr would likely not be deleterious (Fig. 2 and Supplementary Material, Table S2), and this was found to be in agreement with the *in vitro* studies that assessed the effects of these variants on CaSR-mediated ${\text{Ca}}_{\;i}^{2\text{+}}$ signalling (Fig. 4 and Supplementary Material, Fig. S3). However, for other AP2σ variants such pathogenicity and three-dimensional modelling programs were not of value when used alone in correctly predicting the effect of the genetic variants. For example, two of the AP2σ variants, Arg61His and Glu122Gly, were predicted to be deleterious (Figs 2 and 3, Supplementary Material, Table S2), but were instead found to have no effect on CaSR-mediated ${\text{Ca}}_{\;i}^{2\text{+}}$ signalling (Supplementary Material, Fig. S3); while another, Met117Ile, which was predicted to be likely deleterious by pathogenicity programs (Supplementary Material, Table S1) but not three-dimensional modelling (Fig. 3) had impaired signalling (Fig. 4) and was associated with hypercalcaemia in the patient (Table 1). Finally, for the Arg3His and Phe52Tyr AP2σ variants, the pathogenicity and three-dimensional modelling programs gave different predictions (Fig. 2 and Supplementary Material, Tables S1 and S2), and both of these were shown by *in vitro* studies to not result in impaired CaSR signalling (Supplementary Material, Fig. S3), and the Phe52Tyr variant to be associated with normocalcaemia in the patient (Table 1). Thus, it is important to investigate such genetic variants from large-scale sequencing databases with a range of different methods that include population data combined with clinical information, prediction programs, structural models and *in vitro* functional studies, to establish variant pathogenicity.

In conclusion, our studies have identified that non-Arg15 AP2σ mutations may be associated with impaired CaSR-mediated ${\text{Ca}}_{\;i}^{2\text{+}}$ signalling and hypercalcaemia, and that such mutations may cluster at the AP2σ–AP2α inter-subunit interface, and disrupt formation of the AP2 complex with deleterious effects on CaSR function and ${\text{Ca}}_{\;e}^{2\text{+}}$ homeostasis.

## Materials and Methods

### Ethics statement

All clinical data and unique International Classification of Disease-9 (ICD9) codes for each patient were obtained from the electronic health records (EHR) in a de-identified manner through an approved data broker, in accordance with Institutional Review Board approvals (26,30).

### DiscovEHR patient cohort

The study cohort has previously been described in detail (26). In brief, the cohort consisted of Geisinger Health System (GHS) patients within the MyCode Community Health Initiative (26), and whose germ-line DNA underwent whole exome sequencing. Participants were 59% females and 41% males, with a median age of 61 years, and predominantly White (98%) and were enrolled through primary care and specialty outpatient clinics. Exome sequencing was performed as described previously (26). Details of sample preparation, sequencing, sequence alignment, variant identification, genotype assignment and quality control steps, including the setting of allele balance at <0.7, high quality combined allele read depth (AD) of ≥8 reads and per sample genotype quality (GQ) of ≥30, have been described previously (26).

### Online exome and genome sequencing datasets

Two online datasets [ExAc (http://exac.broadinstitute.org/) (24), which includes the 1000 Genomes dataset (http://www.internationalgenome.org), and dbSNP (https://www.ncbi.nlm.nih.gov/projects/SNP/; date last accessed November 2017)] that contain population-based sequencing information from >60 706 unrelated individuals were used.

### Protein sequence alignment and three-dimensional modeling of AP2σ structure

Protein sequences of AP2σ orthologs aligned using ClustalOmega (http://www.ebi.ac.uk/Tools/msa/clustalo/; date last accessed November 2017) (35). SIFT (http://sift.jcvi.org/; date last accessed November 2017) and Polyphen-2 (http://genetics.bwh.harvard.edu/pph2/; date last accessed November 2017) were used to predict the effect of amino acid substitutions (36,37). AP2σ secondary structure was studied using Spider2 (38). AP2σ three-dimensional modelling was undertaken using the reported AP2 structures (Protein Data Bank accession numbers 2XA7 and 2JKR) (10,12). Molecular modelling was performed using the PyMOL Molecular Graphics System (Version1.2r3pre, Schrödinger, LL Pymol) (25).

### Cell culture, constructs and antibodies

Functional assessments of the AP2σ variants were performed using HEK293 cells stably expressing the full-length CaSR (HEK-CaSR), as described previously (3,18). Cells were maintained in DMEM-Glutamax media (ThermoFisher) with 10% fetal bovine serum (Gibco) and 400µg/mL geneticin (ThermoFisher) at 37°C, 5% CO_2_. Wild-type and mutant pBI-CMV4-*AP2S1* expression constructs were generated (using GenBank Accession Number: NM_021575.3), as described (8), and transiently transfected into HEK-CaSR cells using Lipofectamine 2000 (LifeTechnologies). The bidirectional pBI-CMV4 cloning vector was used as it facilitated the co-expression of AP2σ and RFP (8), and site-directed mutagenesis was used to generate the mutant *AP2S1* constructs using the Quikchange Lightning Site-directed Mutagenesis kit (Agilent Technologies) and gene-specific primers (SigmaAldrich), as described (39). The presence of mutations was verified using dideoxynucleotide sequencing with the BigDye Terminator v3.1 Cycle Sequencing Kit (Life Technologies) and an automated detection system (ABI3730 Automated capillary sequencer; Applied Biosystems) (39). Successful transfection was confirmed by visualizing RFP fluorescence using an Eclipse E400 fluorescence microscope with a Y-FL Epifluorescence attachment and a triband 4,6-diamidino-2-phenylindole-FITC-Rhodamine filter, and images captured using a DXM1200C digital camera and NIS Elements software (Nikon) (3,8,18). The expression of AP2σ was also determined by western blot analysis using an anti-AP2σ antibody (Abcam) and expression of calnexin, used as a control, was determined by western blot analysis using an anti-calnexin antibody (Millipore). The western blots were visualized using an Immuno-Star WesternC kit (BioRad) on a BioRad Chemidoc XRS+ system (3).

### Intracellular calcium measurements

The ${\text{Ca}}_{\;i}^{2\text{+}}$ responses of HEK-CaSR cells expressing WT or mutant AP2σ proteins were assessed by a flow cytometry-based assay, as reported (3,8,18). In brief, HEK-CaSR cells were cultured in T75 flasks and transiently transfected 24 h later with 8µg DNA (3). Forty-eight hours following transfection, the cells were detached, resuspended in Ca^2+^ -and Mg^2+^-free Hanks’ buffered saline solution (HBSS) and loaded with 1µg/mL Indo-1-acetoxymethylester (Indo-1-AM) for 1 h at 37°C. Transfected HEK-CaSR cells were incubated with either a 20% aqueous solution of 2-hydroxypropyl-β-cyclodextrin (Sigma) (vehicle) or 10 nm cinacalcet-HCl (Cambridge Bioscience Ltd.), resuspended in vehicle and added to cells prior to flow cytometry analysis (29). After the removal of free dye, cells were resuspended in Ca^2+^- and Mg^2+^-free HBSS and maintained at 37°C. Transfected cells, in suspension, were stimulated by sequentially adding Ca^2+^ to the Ca^2+^- and Mg^2+^-free HBSS to increase the [Ca^2+^]_e_ in a stepwise manner from 0 to 15 mm, and then analysed on a MoFlo modular flow cytometer (Beckman Coulter) by simultaneous measurements of RFP expression (at 525 nm), ${\text{Ca}}_{\;i}^{2\text{+}}$-bound Indo-1-AM (at 410 nm) and free Indo-1-AM (i.e. not bound to ${\text{Ca}}_{\;i}^{2\text{+}}$) (at 485 nm), using a JDSU Xcyte UV laser (Coherent Radiation), on each cell at each [Ca^2+^]_e_, as described (3,18). The peak mean fluorescence ratio of the ${\text{Ca}}_{\;i}^{2\text{+}}$ transient response after each individual stimulus was measured using Cytomation Summit software (Beckman Coulter), and expressed as a normalized response, as described (3,18). Nonlinear regression of concentration–response curves was performed with GraphPad Prism using the normalized response at each [Ca^2+^]_e_ for each separate experiment for the determination of EC_50_ (i.e. [Ca^2+^]_e_ required for 50% of the maximal response). The mean EC_50_ obtained from four to eight separate transfection experiments were used for statistical comparison by using the *F*-test (3,8,18).

## Supplementary Material

Supplementary Material is available at *HMG* online.

*Conflict of Interest statement*. R.V.T. and F.M.H. have received grant funding from NPS/Shire Pharmaceuticals and GlaxoSmithKline for unrelated studies involving the use of calcium-sensing receptor allosteric inhibitors. F.M.H. has received honoraria from Shire Pharmaceuticals and Novartis Pharma AG. R.V.T. has also received grants from Novartis Pharma AG and the Marshall Smith Syndrome Foundation for unrelated studies.

## Funding

This work was supported by: a Wellcome Trust Senior Investigator Award (grant number 106995/Z/15/Z) (RVT); National Institute for Health Research (NIHR) Oxford Biomedical Research Centre Programme (RVT); Wellcome Trust Clinical Training Fellowship (grant number 205011/Z/16/Z) (VJS); and NIHR Senior Investigator Award (RVT) (grant number NF-SI-0514–10091). Geisinger Health System (RM, SBK, GEB); and Regeneron Genetics Center (JGR, JDO). Funding to pay the Open Access publication charges for this article was provided by the Wellcome Trust and National Institute for Health Research (NIHR) Oxford Biomedical Research Centre Programme.

## Supplementary Material

Supplementary Figures and Tables
