# Supplementary material for: Large-scale exome datasets reveal a new class of adaptor-related protein complex 2 sigma subunit (AP2σ) mutations, located at the interface with the AP2 alpha subunit, that impair calcium-sensing receptor signalling
Source: Hum Mol Genet. 2018 Jan 9;27(5):901–11. doi: 10.1093/hmg/ddy010 (PMC5982735; doi:10.1093/hmg/ddy010)
Supplement: Supplementary Figures and Tables [file supplementary_ddy010.pdf]

SUPPLEMENTARY FIGURE 1 Structural model of the AP2 heterotetramer

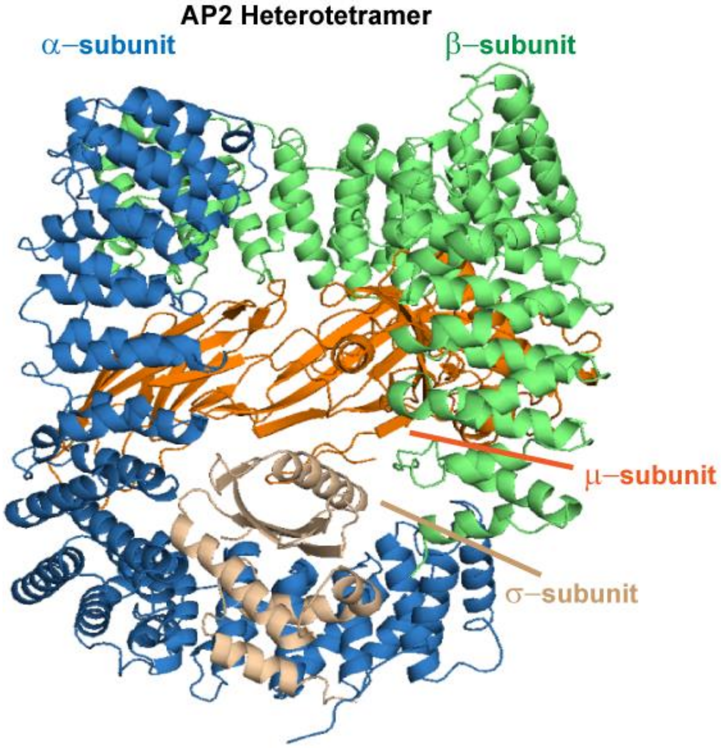

The model shows the α-, β-, μ-, and σ-subunit shown in blue, green, orange and light brown, respectively. Adapted from Kelly *et al*, 2008 (10).

Supplementary Figure 2 Expression of nine AP2σ variant proteins in HEK-CaSR Cells

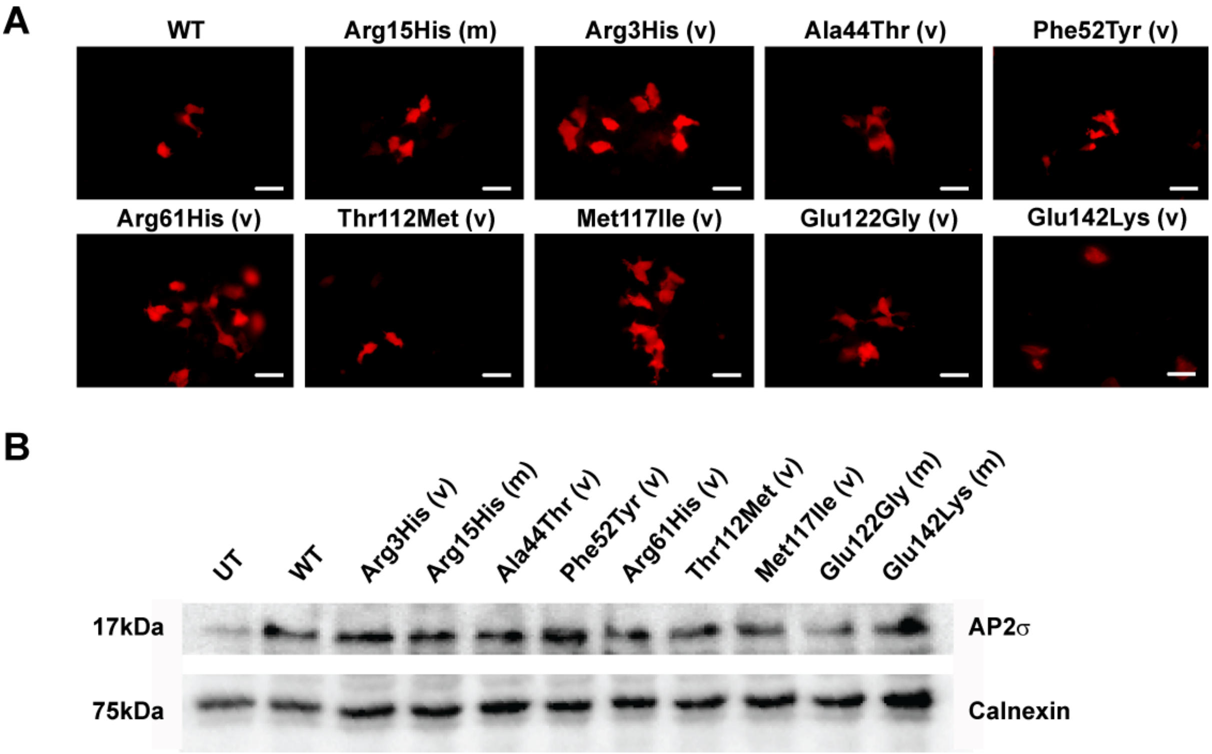

(**A**) Fluorescence microscopy of HEK293 cells stably expressing CaSR (HEK-CaSR) and transiently transfected with WT or variant (v) pBI-CMV4-*AP2S1* constructs. RFP expression in these cells indicates successful transfection and expression by these constructs. Bar indicates 10μm. (**B**) Western blot analysis of lysates from HEK-CaSR cells transiently transfected with WT or variant pBI-CMV4-*AP2S1* expression constructs. The AP2σ protein was overexpressed in transfected cells compared to untransfected (UT) cells in which only endogenous AP2σ was expressed. Calnexin, a housekeeping protein, was used as a control.

SUPPLEMENTARY FIGURE 3 Intracellular calcium responses of cells expressing AP2σ variants (Arg3His, Ala44Thr Phe52Tyr, Arg61His and Glu122Gly) encoded by *AP2S1* exons 2 and 3

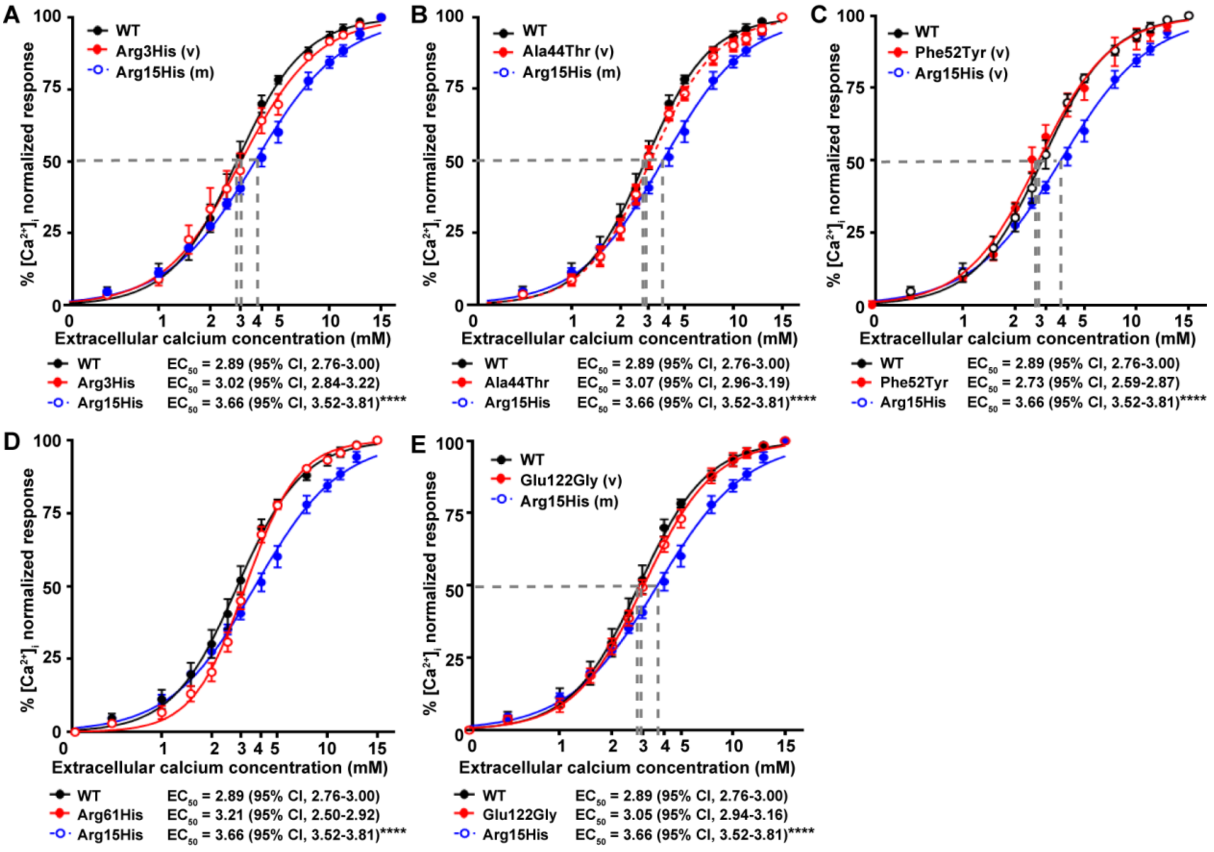

Ca^{2+}_i responses, measured by flow cytometry, to changes in $[Ca^{2+}]_e$ of HEK-CaSR cells transfected with wild-type (WT), or (**A**) Arg3His, (**B**) Ala44Thr, (**C**) Phe52Tyr, (**D**) Arg61His, (**E**) Glu122Gly AP2σ variants (v) (red line), or the FHH3-associated Arg15His mutant (m) (blue line). The Ca^{2+}_i responses to changes in $[Ca^{2+}]_e$ are expressed as a percentage of the maximum normalised responses and shown as the mean±SEM of 4-8 independent transfections. The Arg15His AP2σ variants led to a rightward shift in the concentration-response curve, compared to WT. The AP2σ Arg3His, Ala44Thr, Phe52Tyr, Arg61His and Glu122Gly variant responses were similar to those of wild-type expressing cells. Statistical analysis was performed using the *F*-test. ****p<0.0001 compared to the WT response.

Supplementary Table 1: Non-synonymous variants (N=3) in human *AP2S1* identified in the DiscovEHR cohort^

AA Change[a]	Nucleotide Position	Nucleotide	Allele count	SIFT[b]	PolyPhen[c]
Arg15His	c.44	G>T	3	0	0.434
Phe52Tyr	c.155	G>A	1	0.001	0.443
Met117Ile	c.350	G>T	1	0.026	0.766

^The DiscovEHR cohort represents exome sequencing data from 51,289 individuals. [a]amino acid number according to full-length 142 amino acid protein (GenBank Accession Number: NM_021575.3). All variants were observed in the heterozygous state. [b]SIFT predicts effects of amino acid substitutions based on sequence homology and physicochemical properties, and gives a qualitative (either tolerated or deleterious) and quantitative score (based on the probability that the change is tolerated, i.e. the nearer to 0 the more likely deleterious) score (37). [c]Polyphen-2 predicts effects based on sequence homology, protein databank structures, and protein family database (pfam) annotations (36). It gives a qualitative score of probably damaging, possibly damaging, benign, or unknown, and quantitative scores are based on the probability that the change is damaging, i.e. the nearer to 0 the more benign. A mutation is classified as probably damaging if the score is >0.85, and possibly damaging if the score is >0.15 (36).

Supplementary Table 2: Non-synonymous variants (N=6) in human *AP2S1* identified in the Exome Aggregation Consortium (ExAc) and dbSNP datasets^

AA Change[a]	Nucleotide Position	Nucleotide	Allele Count	SIFT[b]	PolyPhen[c]
Arg3His	c.8	G>A	1	0.56	0.407
Ala44Thr	*c.130*	*C>T*	*1*	*0.34*	*0.014*
Arg61His	c.182	T>C	1	0	0.888
Thr112Met	c.335	C>T	1	0.26	0.476
Glu122Gly	c.365	A>G	1	0	0.95
Glu142Lys	c.424	G>A	1	0	0.624

^ExAc, 1000Genomes and dbSNP contain data from >60,706 unrelated individuals. ExAc includes data from several disease-specific (e.g. diabetes mellitus type 2, inflammatory bowel disease and heart disease patients), and population genetics studies including 1000 Genomes, which has data from 26 populations, and the NHLBI-GO Exome Sequencing Project. The dbSNP dataset is a depository for sequencing information that can be submitted by researchers and contains information on DNA sequence variants identified from the scientific literature. All variants were observed in the heterozygous state. [a]amino acid number according to full-length 142 amino acid AP2σ protein (GenBank Accession Number: NM_021575.3). The dbSNP variant is shown in italics. 1000Genomes did not have any *AP2S1* variants. [b]SIFT predicts effects of amino acid substitutions based on sequence homology and physicochemical properties, and gives a qualitative (either tolerated or deleterious) and quantitative (based on the probability that the change is tolerated, i.e. the nearer to 0 the more likely deleterious) score (37). [c]Polyphen-2 predicts effects based on sequence homology, protein databank structures, and protein family database (pfam) annotations (36). It gives a qualitative score of probably damaging, possibly damaging, benign, or unknown, and quantitative scores are based on the probability that the change is damaging, i.e. the nearer to 0 the more benign. A mutation is classified as probably damaging if the score is >0.85, and possibly damaging if the score is >0.15 (36).

Supplementary Table 3: Synonymous variants (N=21) in human *AP2S1* identified in the Exome Aggregation Consortium (ExAc)^

AA Change[a]	Nucleotide Position	Nucleotide Change	Allele Count
Ile5Ile	c.15	C>T	1
Leu6Leu	c.18	C>T	2
Arg10Arg	c.30	G>A	1
Lys13Lys	c.39	G>A	1
Thr14Thr	c.42	G>T	3
Thr14Thr	c.42	G>A	12
Asp25Asp	c.75	T>C	84
Lys30Lys	c.90	G>A	1
Ile32Ile	c.96	C>T	2
Ala37Ala	c.111	C>T	23
Asp43Asp	c.129	C>T	4
Phe67Phe	c.201	C>T	1
Leu81Leu	c.241	C>T	3
Phe87Phe	C.261	C>T	18
Asn92Asn	c.276	C>T	2
Val98Val	c.294	C>T	2
Asp115Asp	c.345	C>T	1
Leu119Leu	c.357	G>A	1
Thr126Thr	c.378	C>T	1
Thr129Thr	c.387	G>A	4
Leu132Leu	c.396	G>T	1

^ExAc, which contains exome sequencing data from >60,706 unrelated individuals, includes data from several disease-specific and population genetics studies including 1000 Genomes, and the NHLBI-GO Exome Sequencing Project. [a]amino acid number according to full-length 142 amino acid protein (GenBank Accession Number: NM_021575.3).

Supplementary Table 4: Summary table of the effects of AP2σ mutants on the AP2 structure and CaSR signalling

AP2σ mutant	Source	Exon	Location	Evolutionarily conserved	Predicted deleterious by:			Impair CaSR signalling	Associated with hypercalcemia
					SIFT	Polyphen	Structural analysis		
Arg3His	ExAc	2	β1	Yes	?	?	Yes	No	-
Arg15His	DiscovEHR	2	β2	Yes	Yes	?	Yes	Yes	Yes
Ala44Thr	dbSNP	2	α1-β3 loop	Yes	?	No	No	No	-
Phe52Tyr	DiscovEHR	3	β3	Yes	Yes	?	No	No	No
Arg61His	ExAc	3	β4	Yes	Yes	Yes	Yes	No	-
Thr112Met	ExAc	5	α4	Yes	?	?	Yes	Yes	-
Met117Ile	DiscovEHR	5	α4	Yes	Yes	?	No	Yes	Yes
Glu122Gly	ExAc	5	α4-α5 loop	Yes	Yes	Yes	Yes	No	-
Glu142Lys	ExAc	5	α5	Yes	Yes	?	Yes	Yes	-

?, Possible/uncertain. Predictions were based on the classification system used by Polyphen-2 in which the variant was classified as probably damaging if the score was >0.85, and benign when the score was <0.15 (36) -, Unknown.
